# Agronomic gain: Definition, approach, and application

**DOI:** 10.1016/j.fcr.2021.108193

**Published:** 2021-08-01

**Authors:** Kazuki Saito, Johan Six, Shota Komatsu, Sieglinde Snapp, Todd Rosenstock, Aminou Arouna, Steven Cole, Godfrey Taulya, Bernard Vanlauwe

**Affiliations:** a Africa Rice Center (AfricaRice), 01 B.P. 2551, Bouaké 01, Cote d’Ivoire; b Department of Environmental Systems Science, ETH Zürich, Zürich, Switzerland; c Department of Agricultural and Resource Economics, The University of Tokyo, Bunkyo-Ku, Japan; d Department of Plant, Soil and Microbial Sciences, Michigan State University, East Lansing, MI, USA; e Center for International Forestry Research-World Agroforestry, P.O. Box 30677-00100, UN Avenue, Nairobi, Kenya; f International Institute of Tropical Agriculture, P.O. Box 34441, Dar es Salaam, Tanzania; g International Institute of Tropical Agriculture, P.O. Box 7878, Kampala, Uganda; h International Institute of Tropical Agriculture, c/o Icipe, Kasarani, P.O. Box 30772-00100, Nairobi, Kenya

**Keywords:** *Oryza* spp., Agronomy, Productivity, Sustainability

## Abstract

Meeting future global staple crop demand requires continual productivity improvement. Many performance indicators have been proposed to track and measure the increase in productivity while minimizing environmental degradation. However, their use has lagged behind theory, and has not been uniform across crops in different geographies. The consequence is an uneven understanding of opportunities for sustainable intensification. Simple but robust key performance indicators (KPIs) are needed to standardize knowledge across crops and geographies. This paper defines a new term ‘agronomic gain’ based on an improvement in KPIs, including productivity, resource use efficiencies, and soil health that a specific single or combination of agronomic practices delivers under certain environmental conditions. We apply the concept of agronomic gain to the different stages of science-based agronomic innovations and provide a description of different approaches used to assess agronomic gain including yield gap assessment, meta-data analysis, on-station and on-farm studies, impact assessment, panel studies, and use of subnational and national statistics for assessing KPIs at different stages. We mainly focus on studies on rice in sub-Saharan Africa, where large yield gaps exist. Rice is one of the most important staple food crops and plays an essential role in food security in this region. Our analysis identifies major challenges in the assessment of agronomic gain, including differentiating agronomic gain from genetic gain, unreliable in-person interviews, and assessment of some KPIs at a larger scale. To overcome these challenges, we suggest to (i) conduct multi-environment trials for assessing variety × agronomic practice × environment interaction on KPIs, and (ii) develop novel approaches for assessing KPIs, through development of indirect methods using remote-sensing technology, mobile devices for systematized site characterization, and establishment of empirical relationships among KPIs or between agronomic practices and KPIs.

## Highlights

• We define agronomic gain (AG) based on improvement in key performance indicators (KPIs).
• KPIs include productivity, resource use efficiencies, and soil health.
• AG assessment is applied to previous studies with attention to rice in sub-Saharan Africa.
• Challenges in AG assessment in different research process stages are identified.
• Solutions and future research areas in relation to the challenges are provided.

## 1 Introduction

Meeting the future global demand for staple crops produced sustainably is a challenge. With increasing population and per-capita consumption, global demand is expected to grow by 60 % between 2010 and 2050 (Alexandratos and Bruinsma, 2012). As there is little scope for agricultural expansion, growing demand needs to be satisfied through increasing production from the existing area under cultivation. The requisite production growth can be accomplished by reducing the “yield gap” (the difference between potential yield and actual farm yield) and improving genetic yield potential (van Ittersum et al., 2013; Fischer et al., 2014). “Potential yield” is defined as the maximum yield that can be obtained from a crop in a given environment as determined by simulation models with plausible physiological and agronomic assumptions. “Yield potential” is the yield to be expected with the adapted variety, via good management of agronomic and other inputs (Evans and Fischer, 1999). Especially in the Global South, genetic yield potential of existing varieties has not been realized because of abiotic and biotic stresses and poor or suboptimum agronomic practices, resulting in large yield gaps (Fischer et al., 2014; van Ittersum et al., 2016). In addition, various social constraints create yield gaps between female and male farmers, especially in sub-Saharan Africa (SSA) (World Bank, 2014; Kilic et al., 2015). Understanding and alleviating the constraints that create these yield gaps, through enabling actions and agronomic interventions, can help reduce yield gaps (Saito et al., 2013). However, most efforts to reduce yield gaps in the past have been relatively narrow in scope as they do not consider other important concerns of farmers. For example, in areas where yield gaps are low, other issues such as improving resource use efficiencies and increasing resilience to climate shocks could be the primary focus of farmers (Peng et al., 2009; van Oort et al., 2017).

The rate of genetic improvement in the traits of interest within a breeding population over time is referred to as “genetic gain” (Atlin et al., 2017). The traits of interest can differ among breeding programs based on their priorities, but typical traits considered in rice (*Oryza* spp.) breeding programs in SSA include yield, adaptation to climate change including tolerance to abiotic and biotic stresses, and grain quality (Saito et al., 2018; Futakuchi et al., 2021). They do not often include resource (i.e., nutrient and water) use efficiencies, weed competitiveness, and traits that could improve soil fertility (e.g., higher straw production for improving soil fertility). This does not mean that these are not important for rice production, but agronomy could play a key role in improving these trait characteristics. A set of agronomic practices can ensure high productivity and its higher stability, high profitability, high resource use efficiencies, crop biodiversity, minimal emissions of greenhouse gases (GHGs), increased soil health (Dubey et al., 2020), and narrowing productivity.

In order to ensure future food security and meet current needs while conserving land and other resources, sustainable intensification has been put forward as a key approach (Smith et al., 2017). Smith et al. (2017) proposed a wide range of indicators under the domains of productivity, human well-being, and economic, environmental, and social sustainability at different scales (field, farm, and communities). For rice, the Sustainable Rice Platform (SRP) developed “SRP Performance Indicators” for environmental, economic, and social sustainability (SRP, 2019). The use of such indicators has been lagging behind the theory and are not uniform among a wide range of organizations (Wyn Ellis, Executive Director, SRP, personal communication). This paper proposes to separate direct improvement in a few key performance indicators (KPIs) as an entry point for agronomic intervention from their 'holistic evaluation', that require contributions from other dimensions (e.g., education, women’s empowerment). Based on our understanding, KPIs that specifically focus on what agronomic practices can deliver do not exist. Such KPIs could help rapid, efficient, and robust monitoring of both development of agronomic practices and their scaling in agricultural research-for-development programs.

The KPIs proposed in this paper were identified based on discussion among colleagues in Excellence in Agronomy 2030 initiative (EiA 2030), including 10 CGIAR research centers (AfricaRice, CIAT, CIMMYT, CIP, ICARDA, ICRAF, ICRISAT, IFPRI, IITA, and IRRI), and external partners. These are linked to all the impact areas proposed in CGIAR 2030 Research and Innovation Strategy (CGIAR System Office, 2021), consisting of (i) nutrition and food security, (ii) poverty reduction, livelihoods and jobs, (iii) gender equality, youth, and social inclusion, (iv) climate adaptation and greenhouse gas reduction, and (v) environmental health and biodiversity (Table 1). Given that a common set of KPIs is not available in the framework of EiA 2030, we are proposing these KPIs to assess impact of agronomic innovations in different geographies and across crops as a standardized monitoring and evaluation tool. Aligning with this special issue, we focus on assessing the impact of rice agronomic innovations with particular attention to rice in SSA as a case study. Rice is one of the most important staple food crops and plays an essential role in food security in this region (van Oort et al., 2015; van Ittersum et al., 2016). It has been frequently reported that large yield gaps exist, and sub-optimal agronomic practices are one of major constraints to increasing rice productivity and resource use efficiencies in this region (Niang et al., 2017; Tsujimoto et al., 2019).

**Table 1:** Key performance indicators, their typical units, and their linkages with impact areas of the One CGIAR research and innovation strategy 2030 (CGIAR System Office, 2021).

KPI	Detailed indicator/description	Typical unit	Link with impact areas*
Productivity	Crop yield	kg/ha	(i), (ii), (iii), (iv)
	Temporal and spatial variation of crop yield (e.g., coefficient of variation)	%	
	Profitability or cost-benefit balance, which is calculated by (harvested product price × change in yield with improved agronomic practices in comparison with current farmer’s yield) – (changes in production cost** induced by the use of improved agronomic practices)	$/ha	(i), (ii), (iii)

Resource use efficiency	Nutrient-use efficiency (e.g., nitrogen, phosphorus) (in other word, partial factor productivity of applied nutrient)	kg yield/kg nutrient input	(i), (ii), (iii), (iv), and (v)
		kg nutrient in yield/kg nutrient input	
	Water productivity	kg yield/l water (rainfall + irrigation)	(iii), (iv), and (v)
	Labor productivity	kg yield/person-day	(ii) and (iii)

Soil health	Soil organic carbon (SOC)***	g/kg	(ii), (iii), (iv), and (v)

* Impact area: (i) nutrient, health & food security, (ii) poverty reduction, livelihood, & jobs, (iii) gender equality, youth & social inclusion, (iv) climate adaptation & GHG reduction, and (v) environmental health & biodiversity.

** If improved fertilizer management practices are introduced, we consider difference in fertilizer cost per area between improved practices and control for production cost (Saito et al., 2015).

*** Temporal change in SOC will be monitored by agronomic practice and site characteristics.

The objectives of this paper are to: (1) define agronomic gain and provide a conceptual framework for its assessment; (2) describe different approaches for assessing agronomic gain at different stages of the research process on agronomic innovations from benchmarking of current situations to development and piloting of innovations, and impact assessment of innovations across different scales from field to subnational and national levels; (3) identify the challenges in assessment of agronomic gain; and (4) suggest further research areas for assessing the agronomic gain for rice in SSA in different stages of the research process for agronomic innovations at different scales.

## 2 Concepts

### 2.1 Prioritization of agronomic gain key performance indicators

Here, we define “agronomic gain” as the improvement in key performance indicators (KPIs) related to sustainability, including aspects of productivity and environment, through a specific single or combination of agronomic practices under specific environments and social contexts. KPIs consist of productivity, resource use efficiencies, and soil health (Table 1). Productivity includes yield, its stability, and profitability, whereas resource use efficiencies include labor productivity, nutrient use efficiency such as nitrogen use efficiency (NUE) and phosphorus use efficiency (PUE), and water productivity. Justification for selection of these KPIs is as follows:

• An increase in food security and market surplus requires an increase in the yields of smallholder farmers and a reduction in their risks to climate shocks through an increase in yield stability;
• Increased household income to afford food expenses, health services and education and to invest in the farm requires increased profitability or cost-profit balance;
• Improved nutrient-use efficiency with agronomic interventions leads to improved yield or decreased input costs, higher profitability, increased food security, less nutrients lost to the environment, reduction of emissions of GHG from fields (especially for nitrogen), and decreased energy consumption from the production and transportation of fertilizers;
• Through improved water use efficiency, savings in irrigation or rain water can be used for other important purposes;
• Increased labor productivity leads to increased profitability, more time to spend on other activities, increased attractiveness of crop production and increased willingness to invest in the farm; and
• Building up soil carbon content (SOC) can lead to increased resilience to climatic shocks, decreased input cost, and a reduction in energy consumption from the production and transportation of chemical inputs.

Their linkages with the five impact areas of the One CGIAR research and innovation strategy 2030 are listed in Table 1 (CGIAR System Office, 2021). All proposed KPIs are assessed for having systematic product profiles (i.e., improved agronomic practices) and analyzing their tradeoff and synergistic effects when introducing improved agronomic practices (Snapp et al., 2019). However, we recognize that the local context will determine which KPIs are relevant for use based on the needs of different end users. There could be need to accept some trade-offs among KPIs in the short term, while moving towards positive synergies among KPIs in the long term. If different end users identify other important indicators, they can be added. For example, if there is specific soil-related problems (e.g., acidity, salinity, poor soil structure), soil parameters related to such problems can be included in the set of KPIs. Here, we do not propose to aggregate multiple indicators into a single index for agronomic gain because the application of weights for different indicators is subjective and a simple adding is not advantageous either.

### 2.2 Definition of agronomic gain

The agronomic gain in KPIs is the difference in the given indicators between improved agronomic practices and the control (or current) practices that farmers use when cultivating the same crop variety. For example, for yield, actual agronomic gain in yield equals yield under improved agronomic practices minus yield under current farmers’ practices. Thus, this gain is determined by the interaction of G(enotype) x E(nvironment) x M(anagement). Hence, genetic gain and agronomic gain are both characterized by spatial and temporal variation, but management is the factor that distinguishes the agronomic gain from the genetic gain.

Agronomic gain can be divided into potential and actual gain. For example, as is the case for yield, the yield gap between potential yield and actual yield may be potential agronomic gain, whereas the yield gap between water-limited potential yield and actual yield may be water-limited potential agronomic gain (van Ittersum et al., 2013; van Oort et al., 2015). Potential yield and water-limited potential yield can be determined by crop simulation models. As they were defined by van Oort et al. (2015), we do not describe them in this paper. From potential yield, water-limited potential yield, and actual yield, potential or water-limited absolute and relative agronomic gain in yield are calculated as follows:

(1) Potential or water-limited absolute potential agronomic gain in yield = (Y_p_ or Y_w_) – Y_a_

(2) Potential or water-limited relative potential agronomic gain in yield = [1 – Y_a_/(Y_p_ or Y_w_)] × 100

where Y_p_ is potential yield, Y_w_ is water-limited potential yield, and Y_a_ is actual yield obtained under farmer’s fields.

The actual agronomic gain in yield can be determined on the ground and at scale. Thus, we define that it is the attained yield effect of a single or a combination of agronomic practices obtained on-farm, which is dependent on the crop type and crop variety (i.e., G) and the location (i.e., E). It can be described by the following equation:

(3) Actual agronomic gain in yield = Y_i_ – Y_a_

Where Y_i_ is yield under improved agronomic practices.

When agronomic gain is assessed in farmers’ fields over time, we need to consider not only mean agronomic gain, but also the distribution of agronomic gain across farmers, disaggregated by the sex of the farmer. For example, even if agronomic gain is observed on average for the target male and female farmers, gain may be observed in less than 50 % of these farmers. Thus, the distribution of agronomic gain should be evaluated through use of mean, coefficient of variation, skewness, and probability analysis, and disaggregated by sex, to identify for who or under which conditions higher or lower agronomic gain is observed (Laborte et al., 2012; Sharma et al., 2019).

### 2.3 Agronomic vs genetic gain

**Fig. 1:**
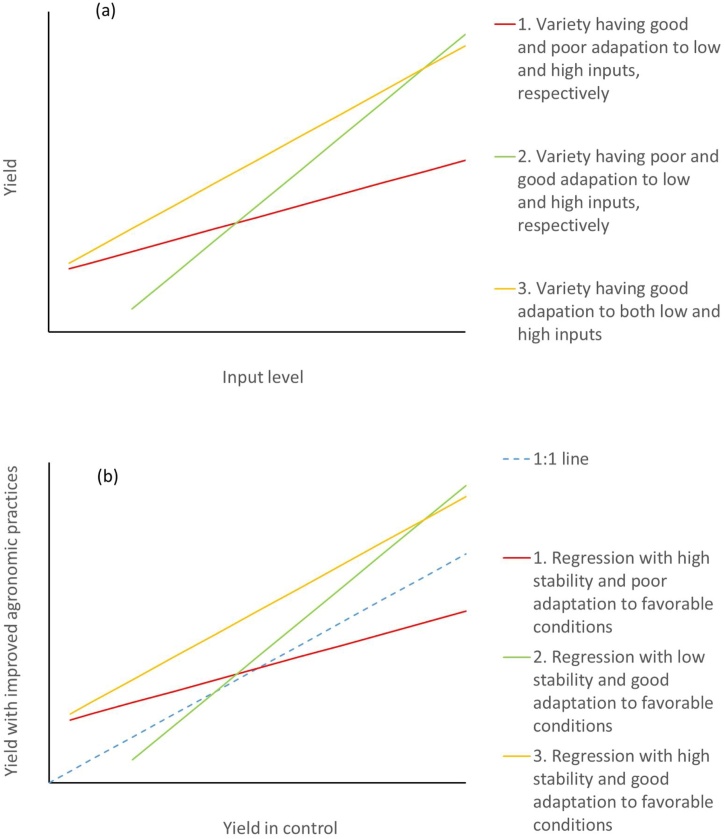
Simple illustration of (a) yield of different varieties at different input levels, and (b) yield stability of improved agronomic practices against control at different yield levels.

Here, we distinguish the contribution of agronomic practice from genotype (variety) effect. This can be done in factorial experiments, by including both variety and agronomic practice as factors. An example of a two-way analysis of variance model in randomized control block design is as follows:

(4) Y_ijk_ = μ + G_i_ + Mj + (GM)_ij_ + e_ij_

where Y_ijk_ is the given indicator (e.g., yield) of a plot, μ is the experiment mean, G_i_ is the effect of the ith variety, Mj is the effect of the jth agronomic practice, (GM)_ij_ is the interaction of the ith variety and the jth agronomic practice, and e_ij_ is the residual. When variety × agronomic practice interaction is significant, the contribution of the improved agronomic practice to the given indicator depends on variety. For example, an improved agronomic practice with high level of input(s) will only be effective for input-responsive varieties (e.g., Saito and Futakuchi, 2009). Fig. 1(a) is a simple illustration of yield of different varieties at different input levels. Regression (1) shows variety having good adaptation to low input, but low adaptation to high input. In contrast, regression (2) shows variety having poor adaptation to low input, but high adaptation to high input. The desirable regression is (3), which has high adaptation to both input levels.

### 2.4 Agronomic gain in relation to environmental conditions

There may also be an agronomic practice × environment interaction, where agronomic gain is different across diverse growing environments (temporal and spatial dimensions). Thus, it is essential to assess the impact of improved agronomic practices on agronomic gain in a wide range of growing conditions in the target environment to quantify agronomic practice × environment interaction and stability. Ideally, improved agronomic practices would give positive impact under both favorable and unfavorable conditions. Fig. 1(b) is a simple illustration of yield stability of improved agronomic practices against control at different yield levels. Regression (1) shows good adaptation to low-yielding environments, but low adaptation to high-yielding environments. In contrast, regression (2) shows poor adaptation to low-yielding environments, but high adaptation to high-yielding environments. The desirable regression is (3), which has a combination of high stability and high adaptation to high-yielding environments. If low-yielding conditions are associated with climate shocks, the improved agronomic practices provide resilience-reduced yield reduction by climate shock. When there is an agronomic practice × environment interaction, it is important to define target environment where agronomic practices can work well for scaling. In case of long-term panel data sets or national statistics, season-to-season variability and stability of agronomic gain could be assessed through trend analysis.

### 2.5 Agronomic gain in relation to the social context

There may also be an agronomic practice × social context interaction, where agronomic gain is different across diverse social contexts given certain social and gender constraints that different farmers face (Achandi et al., 2018). The use of an improved agronomic practice may result in good performance for male farmers who have adequate time and resources to implement the practice, yet result in sub-optimal performance for female farmers who struggle to adequately implement the practice given women’s unpaid (domestic) work activities and limited decision-making powers to re-allocate resources to hire labor to supplement their limited labor contributions (Kinkingninhoun et al., 2020). Thus, it is essential to assess the impact of improved agronomic practices on agronomic gain in different social contexts and within different household types (Arouna and Akpa, 2019). Ideally, improved agronomic practices would give positive impacts across different social contexts, yet gender productivity gaps are pervasive in certain contexts (e.g., World Bank, 2014). Accordingly, there is need to collect sex-disaggregated data at plot level to determine if gender gaps in KPIs exist as well as the determinants of these differentials.

## 3 Stages of the research process for assessment of agronomic gain

Agronomic gain can be determined for the different stages of the research process: discovery, proof of concept, pilot, and scaling (Table 2). The discovery stage attempts to quantify potential agronomic gain with improved agronomic practices and target environment for investment and interventions. Research activities include baseline surveys, yield gap assessments, participatory diagnostic surveys or trials, ex-ante analysis of promising and alternative innovations using crop simulation models. Here, we also consider meta-analysis as it can identify promising agronomic practices for their testing at the target environment. The proof of concept stage involves testing of improved agronomic practices on station and/or farms to assess agronomic gain. Agronomic trials include multi-factorial trials and multi-environment trials. The pilot stage should demonstrate technology readiness for scaling in both technical and socio-economic aspects, and develop strategies for scaling. Agronomic practices are piloted with farmers through a participatory approach under close supervision by scientists and extension workers at field level (but not at small plot level). This piloting could help scientists fine-tune the innovations and make them adapted to local conditions and especially ensure the innovations are gender-responsive, before scaling of the innovations (Achandi et al., 2018; Zossou et al., 2020). At the late pilot stage, the innovations can be disseminated by development projects. This stage also includes ex-ante impact assessment studies led by economists by applying advanced methodologies to evaluate improved agronomic practices for generating science-based evidence on potential adoption of the innovations and their potential development impact (e.g., Arouna et al., 2021), for example, taking into account the CGIAR five impact areas highlighted above. The scaling stage is the process of replicating and/or adapting innovations across large geographies and populations for adoption and impact. At the early scaling stage, the innovation can have large adoption and impacts at scale beyond the development projects. Here, panel studies and ex-post adoption and impact assessment are key tools for providing science-based evidence. The science-based evidence is expected to influence policies and enhance dissemination of the innovations by private and public sectors for wide-scale adoption.

**Table 2:** Description of different approaches in the four stages of the research process and challenges and considerations for assessing agronomic gain.

Stage	Approach for assessing agronomic gain	Rationale and characteristic	Scalability of assessment	Challenges and considerations
Discovery	• Global Yield GAP and Water Productivity Atlas • Ex-ante analysis of promising and alternative agronomic practices using crop simulation models	• Potential agronomic gain assessed by using the bottom-up approach together with use of crop simulation models at sub-national, national levels.	• Possible to assess potential agronomic gain in a few indicators (yield, its stability, and water productivity) at multiple scales from field to national level.	• Data availability for crop modeling and yield gap assessment. • Resource use efficiency (except for water productivity) cannot be determined.
	• Baseline and diagnostic surveys targeting male and female farmers at individual farm (or plot) level	• The surveys allow to determine all KPIs, whereas above approach can determine a few indicators.	• Data are typically collected through farm records or recall survey at field or individual farm level. These surveys can be done at sub-national level.	• Data collected through in-person interviews are often unreliable. • Difficult to quantify impact of agronomic practices solely as variety × agronomic practice (genotype-by-management; G × M) interaction tends to be high.
	• Meta-analysis	• This helps identifying potential agronomic gain by promising agronomic interventions for testing.	• Agronomic gain can be assessed by use of data from previous multiple studies.	• Previous studies often focus on a few indicators only.
Proof of concept	• Testing of improved agronomic practices in on-station and/or on-farm trials	• This approach examines the effect of the agronomic practice and its interaction with season and location, as well as variety on KPIs.	• Agronomic gain can be assessed at plot level in field experiments. • Data are typically collected through field measurement at plot level. • These trials can be done at sub-national level.	• Monitoring of some indicators (e.g., water productivity and nutrient use efficiency) should be done at this stage because it is difficult to determine them in later stages. • Genotype × management × environment trials are fundamental to quantify impact of M on agronomic gain.
Pilot	• Participatory on-farm trials	• This approach can help scientists fine-tune their prototype innovations and make them adapted to local conditions, before scaling.	• Agronomic gain can be assessed at individual plot or field level. • Data are typically collected through field measurement or farm records/household recall survey at plot or field level. • These trials can be done at sub-national level.	• Monitoring of agronomic practices is needed. • Identifying suitable approaches for participatory testing is needed to make sure that improved agronomic practices will be tested with different social groups.
	• Ex-ante impact assessment study	• The impact assessment study helps evaluating the potential adoption of improved agronomic practices and their impact on KPIs.	• Data are typically collected through farm records or household recall survey at field or individual farm level. • These surveys can be done at sub-national level.	• Data collected through field measurement is not possible, and data through in-person interviews are often unreliable. • Research is needed for developing novel approaches for assessing KPIs, through development and introduction of remote-sensing technology or mobile devices that can estimate KPIs (e.g., yield, soil health) and identification of empirical relationships among KPIs or between agronomic practices and KPIs for indirect assessment. • Information on enabling conditions (e.g., access to land, credit, crop insurance, inputs, mechanization, training, market) and constraining factors (e.g., harmful gender norms) is also essential for identifying reasons behind farmers’ good and/or sub-optimal agronomic practices.
Scaling	• Panel studies and ex-post adoption and impact assessment	• These provide science-based evidence for actual adoption of agronomic practices and agronomic gain at household and plot levels.	• Data are typically collected through farm records or household recall survey at field or individual farm level. • These surveys can be done at sub-national level.	• Same as three bullet points at ex-ante impact assessment study in pilot stage. • Long-term efforts are needed to have impact at scale. • Agronomic practices often consistent of various component technologies and farmers often gradually take up components in sequence. Furthermore, they are often modified by farmers; thus, it is difficult to assess their adoption. • Climate variability can easily mask impact of M especially in rainfed systems (this also applies to other stages).
	• Use of data from sub- or national statistics	• This approach enables to quantify long-term trend in KPIs at sub-national or national level.	• Data are collected at sub- or national level.	• Data are often unavailable or unreliable especially in the Global South. • Without other information related to variety replacement and change in agronomic practices, quantification of agronomic gain is not possible.

## 4 Approaches to assess agronomic gain and examples of agronomic gain

In this section, we introduce different approaches for assessing agronomic gain in different stages, based on Table 1, Table 2, and show examples of agronomic gain assessed by these approaches.

### 4.1 Global Yield Gap and Water Productivity Atlas

Potential agronomic gain in yield can be assessed by using the bottom-up approach together with use of crop simulation models, developed by the Global Yield Gap and Water Productivity Atlas (GYGA) (www.yieldgap.org). This atlas considers potential and water-limited agronomic gain in yield and water productivity under farmers’ conditions. Detailed approach is described on the website and papers (e.g., van Ittersum et al., 2013). Here, we briefly show agronomic gain reported for rice, and identify areas for target environment for investment and interventions as example (e.g., high potential agronomic gain in yield). Up to 4 March 2021, the GYGA covered 88 % of the global rice. In both irrigated and rainfed rice, potential absolute and relative agronomic gain in yield varied widely across regions and countries (Table 3). Generally, rainfed rice tended to have higher potential absolute and relative agronomic gain in yield. Irrigated rice in Southeast and South Asia, SSA, and Brazil, and rainfed rice in Brazil had higher potential absolute agronomic gain in yield (> 5 t/ha, >50 %) than other regions (Table 3). Although potential absolute agronomic gain in yield is smaller in rainfed rice in SSA countries than Brazil, potential relative agronomic gain in yield is similar.

**Table 3:** Absolute and relative potential agronomic gain in yield (t/ha) and temporal variation of potential or water-limited yield (CV; %) in selected countries. Data are from the Global Yield Gap and Water Productivity Atlas (GYGA — www.yieldgap.org; van Ittersum et al., 2016). Data were downloaded on 4 March 2021. Dark and light green indicate low potential/water-limited absolute or relative agronomic gain in yield, and low CV, whereas red and orange indicate high potential/water-limited absolute or relative agronomic gain in yield and high CV. (For interpretation of the references to colour in this table legend, the reader is referred to the web version of this article.)

*Potential absolute agronomic gain in yield of irrigated rice (t/ha), and water-limited absolute agronomic gain (t/ha) in yield of rainfed rice.

**Potential relative agronomic gain in yield of irrigated rice (%), and water-limited relative agronomic gain (%) in yield of rainfed rice.

***Empty cells indicate that data were not available from GYGA website.

The GYGA provided with yield stability that calculated by year-to-year variation in potential yield or water-limited potential yield, as well as actual yield (van Oort et al., 2015) (Table 3). Rainfed rice in SSA had lower yield stability. This result clearly indicates that rainfed rice in SSA countries especially requires great attention for stabilizing rice production. If climate risk is too high, farmers will not invest in their crop (van Ittersum et al., 2013). In such circumstances, enhancing resilience to climate risk through introduction of irrigation systems, on- and/or off-farm diversification, crop insurance, and/or use of weather forecasting for optimizing production is more important than focusing on the introduction of agronomic practices for enhancing agronomic gain in yield only.

### 4.2 Ex-ante analysis of promising and alternative agronomic practices using crop simulation models

Crop simulation models can be used for ex-ante analysis of promising and alternative agronomic practices using current climate data and future climate scenarios to inform the research agenda. For example, in irrigated rice systems in the Senegal River valley, van Oort et al. (2016) developed a model to generate, compare, and visualize opportunities for single, double, and triple cropping systems consisting of irrigated rice and optionally a vegetable. Most promising options to increase rice production are through shifting the sowing date to facilitate double cropping, adoption of medium-duration varieties, and breeding for cold-tolerant varieties.

### 4.3 Baseline and diagnostic surveys

A limitation of use of crop modeling approach for assessing potential gains is that, apart from yield, only a few indicators can be determined. An alternative approach is to conduct or use farm baseline survey data to calculate potential agronomic gain in the given indicators as following for yield.

(5) Potential absolute agronomic gain in yield = Y_e_ – Y_a_

Whereas Y_e_ is best farmers’ value (average of the upper 10th percentile) or value from field trials for setting priorities for interventions (van Ittersum et al., 2013; Saito et al., 2017). Recently, AfricaRice and its partners have used Y_e_ to quantify potential absolute agronomic gain in yield in SSA (Tanaka et al., 2015; Niang et al., 2017; Paresys et al., 2018; Tanaka et al., 2017; Senthilkumar et al., 2020; Dossou-Yovo et al., 2020). However, other KPIs were rarely reported in this region. In Asia, Devkota et al. (2019) assessed potential agronomic gain in yield, profit, labor productivity, nitrogen use efficiency (NUE), phosphorus use efficiency (PUE), and water productivity. Here, the difference between the 10 % highest-performing farms (mean of top decile) and the mean-performing farms can be exploitable total gains, which include effects of genotype (G), agronomic practice (M), and G × M interaction (Eq. (1)). The ranges in exploitable total gains in profit, labor productivity, nitrogen use efficiency (NUE), phosphorus use efficiency (PUE), and water productivity are 24–42 %, 36–82 %, 12–32 %, 11–20 %, 1–29 %, and 12–42 %. Exploitable total gain in profit is the largest among them and requires cost-saving innovations. Limitation in this type of survey is that highest-performing farms may not represent what is biophysically possible, as they are also affected by socioeconomic factors (Devkota et al., 2019). Nevertheless, the assessment using highest-performing farms contributes to a better understanding of what could be achieved, based on location-specific environmental and socioeconomic conditions with current agronomic practices (Stuart et al., 2016).

Diagnostic surveys through field observation, crop cut, and interview, or trials such as nutrient omission trials have also been used in recent studies to identify extent of agronomic gain and factors affecting the gain (e.g., yield gap decomposition studies), and identify key agronomic intervention areas (Silva et al., 2017, 2018; Niang et al., 2017, 2018; Tanaka et al., 2017; Rodenburg et al., 2019; Saito et al., 2019; Tsujimoto et al., 2019; Dossou-Yovo et al., 2020). In addition to data from surveys, data from nutrient response trials or water management trials could provide with maximum level of nutrient use efficiency or water productivity (Zwart and Bastiaanssen, 2004; Tsujimoto et al., 2019). Finally, baseline and diagnostic surveys can easily be administered with female and male farmers, ideally at individual plot level, to ascertain gender differences in agronomic gain (e.g., Ali et al., 2016; Kinkingninhoun et al., 2020). Often, these data are not collected, which reflect a bias towards understanding the technical issues that influence agronomic gain rather than the use of an approach that considers both the technical and social constraints that limit the achievement of such gains.

### 4.4 Meta-analysis

Meta-analysis is one of statistical approaches to combine the results from multiple studies in an effort to increase power, improve estimates of the size of the effect, and/or to analyze inconsistency of results across the studies and identify under which conditions the given agronomic practices can deliver better agronomic gain. This approach could identify potential agronomic practices for testing in the target environment. Table 4 shows selected papers dealing with meta-analysis of rice agronomic practices in different regions, including SSA. Enhanced-efficiency nitrogen fertilizers and site-specific nutrient management practices (SSNM) had positive impact on agronomic gain in yield. Nitrogen use efficiency was improved with slow-released N fertilizer, and SSNM. Water-saving technologies and alternate wetting and drying (AWD) reduced rice yield, but improved water productivity. In contrast, direct-seeded rice under wet tillage increased rice yield and profitability, and reduced water use, indicating agronomic gain in water productivity.

**Table 4:** Overview of selected publications on rice agronomy meta-analyses (Carrijo et al., 2017; Chakraborty et al., 2017; Chivenge et al., 2021; Linquist et al., 2013).*

*A literature survey of peer-reviewed publications was carried out with the words “meta-analysis” and “rice” using Google Scholar (Google Inc., Mountain View, CA, USA) for articles published online before November 2019. We selected publications dealing with studies on the effect of agronomic practices only. In addition, one paper (Chivenge et al., 2021) was added in March 2021.

### 4.5 Testing of improved agronomic practices in on-station and on-farm trials

Once a potential agronomic practice (concept) is identified, agronomists typically conduct field trials for several seasons in multiple locations within the target environment to examine the effect of the agronomic practice and its interaction with season and location. They may also add variety treatment to examine G × M interactions. There has been a great number of publications related to this type of work. These include both on-station and on-farm trials. Agronomic gain is best assessed through comparison between improved agronomic practices and farmers’ practices (both female and male) in multi-environment trials. In addition to such on-farm, multi-environmental trials, long-term trials should be considered for assessing impact of improved agronomic interventions on long-term agronomic gain in KPIs and their stability (e.g., Haefele et al., 2002; Bado et al., 2010). For example, Bado et al. (2010) reported that continuous cultivation of irrigated rice with balanced fertilization on submerged soils in Senegal River Valley slightly increased SOC (Table 5). Similar results were also obtained in the Philippines (Pampolino et al., 2008).

**Table 5:** Agronomic gain observed in selected studies on rice in sub-Saharan Africa (Bado et al., 2010;de Vries et al., 2010; Djaman et al., 2018; Haefele et al., 2000, 2001; Ibrahim et al., 2021; Krupnik et al., 2012; Rodenburg et al., 2015; Saito et al., 2015; Segda et al., 2005).

It is noted that within KPIs, impact of agronomic interventions on nutrient use efficiency with unit of kg nutrient in yield/kg nutrient input and water productivity can be mainly measured at this proof of concept stage, as assessment of these requires measurement of nutrient concentration or irrigated water and they cannot be easily determined in later stages, when data are mainly collected through farm records or household recall survey.

Here, we summarize agronomic gain observed in selected studies on rice conducted in the last two decades in SSA with focus on integrated crop management, site-specific nutrient management, alternate wetting and drying (AWD) as a water-saving practice, weed management as a labor-saving practice (Table 5). Three studies on integrated management practices for irrigated rice systems in Table 5 clearly showed increase of yield and profitability. Since the 1990s, rice agronomists have worked on site-specific nutrient management practices, and agronomic gain in yield, profitability, N-use efficiency from those practices in on-farm trials have been quantified and reported (e.g., Segda et al., 2005; Saito et al., 2015). Saito et al. (2015) reported that higher agronomic gain in yield with improved nutrient management practices was weakly associated with lower yield in farmers’ practices. Similarly, Sharma et al. (2019) reported that low-yielding farmers had better gains in net income with improved nutrient management practices than high-yielding farmers in India. These two studies clearly indicate that agronomic gain should be assessed not only by average gain, but also by distribution of the gains. AWD clearly showed a reduction of water use without a significant yield reduction, and de Vries et al. (2010) showed its positive impact on water productivity. These results confirm the findings of meta-analysis (Table 4, Carrijo et al. (2017)). Furthermore, it has been reported that AWD can also reduce global warming potential (e.g., Tirol-Padre et al., 2018), although it has not yet been quantified in SSA. Use of rotary weeders and herbicide application significantly reduced weeding time without yield penalty, indicating improvements in labor productivity. Although we reviewed selected papers only, we found that most papers only consider one to three KPIs.

### 4.6 Participatory on-farm trials

The pilot stage should demonstrate the technology’s readiness for scaling from both a technical and socioeconomic perspective, and develop a strategy for effective scaling. This requires science-based evidence for adoption of the innovations and their potential impacts. Once the problems are jointly identified and promising agronomic practices are well validated by researchers, these agronomic practices are piloted using participatory research approaches. Through an iterative learning phase conducted with systematized feedback from farmers, the fine-tuning of innovations can be carried out (Krupnik et al., 2012). This is essential, to adapt agronomy to local conditions, before scaling out. For example, in the Senegal River Valley, researchers collaboratively developed ‘farmer adapted practice’ to modify recommended practices to fit farmers’ needs and assets (Krupnik et al., 2012).

There is a wide diversity about the scope and nature of farmer participation in the implementation of participatory research with farmers (Sanginga et al., 2006). But, for participatory on-farm trials to assess agronomic gain, we could typically consider following two types (Franzel and Coe, 2002):

• Trials are researcher-designed but farmer-managed, e.g., farmers agree to implement a common design. It is useful to get farmer feedback on specific prototypes or for conducting economic analyses.
• Trials are farmer designed and managed where farmers can experiment on their own. The objective of this type of trial is to assess farmer innovation and acceptability.

It is noted that trials that are researcher-designed and managed belong to the proof of concept stage. The choice of trials depends on the objective of the trials. Researcher and farmer led trials can be conducted at both individual level and community level, depending on the type of agronomic interventions (Sanginga et al., 2006). For example, introduction of water and crop management options at watershed level or irrigation scheme levels requires community level collective action (Poussin et al., 2006; Arouna and Akpa, 2019).

### 4.7 Ex-ante impact assessment study

In pilot stage, impact assessment studies are essential for evaluating the improved agronomic practices. However, Yamano et al. (2016) reviewed impact assessment studies on rice over 10 years (2006–2015) in the world and found that such studies on agronomic practices are especially limited in number and area coverage; there has been no study dealing with soil health; and none of the studies applied advanced methodologies for impact assessment. For example, Lampayan et al. (2015) reviewed studies in Bangladesh, the Philippines, and Vietnam, and compared yields and economic returns by using a “with and without” analysis between AWD users and non-users and a “before and after” analysis among AWD adopters. The results showed higher net income (47–229 US$/ha) from the use of AWD without yield penalties (−0.1 to 0.7 t/ha yield increase) (see Table 5 in Yamano et al., 2016). Limitations of this analysis are discussed by Yamano et al. (2016). Here, one could ask how we can differentiate agronomic gain from variety effect in impact of AWD. As treatment and control farmers were not randomly selected before introduction of AWD, it might be possible that some farmers adopting a specific variety might have adopted AWD more than those who did not use the variety. Thus, it is not possible to separate these effects between AWD and variety interaction.

One methodology for impact assessment, randomized control trials (RCTs), has become the “gold standard” among economists in recent years. Under RCTs, farmers are randomly selected and assigned into two groups: treatment and control. Farmers in the treatment group receive an improved agronomic practice, whereas farmers in the control group do not. After one cropping season, outcome (here, agronomic gain) for the two groups is compared to identify the impact of adopting the new technology. The main assumption is that, because farmers in both groups are selected randomly, the two groups are comparable on average. Implementation of RCTs requires collaboration between agronomists and impact assessment specialists. Recently, with such a collaboration, Arouna et al. (2021) assessed impact of digital decision support tool “RiceAdvice” as one of SSNM practices on agronomic gain in yield and profitability in northern Nigeria. The households who were just given the advice generated by RiceAdvice increased their yield by 7 % and increased their profit by 10 %. On average, the advice increased yield without increasing the overall quantity of fertilizer used, indicating that nutrient use efficiency was also improved.

### 4.8 Panel studies and ex-post adoption and impact assessment

**Fig. 2:**
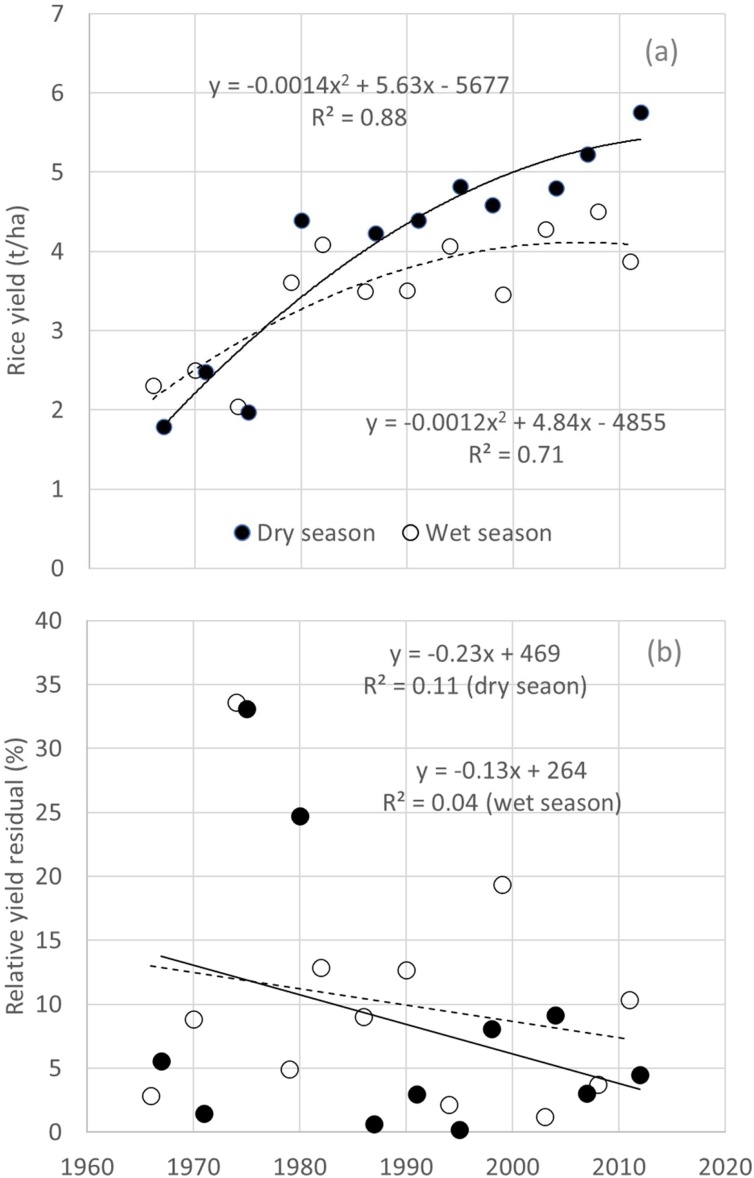
Relationship between year from 1966 to 2012 and (a) yield and (b) relative yield residuals of rice grown in dry (solid curve or line) and wet (dotted curve or line) seasons in central Luzon, the Philippines.

In scaling stage, panel studies and ex-post adoption and impact assessment are key tools for providing science-based evidence on agronomic gain (Yamano et al., 2016). As there is no ex-post adoption and impact assessment study on recently developed rice agronomic practices at large scale in SSA, we show assessment of agronomic gain using data from a panel survey on irrigated lowland rice in the dry season of 1966–2012 in central Luzon, the Philippines (Moya et al., 2015), where yield increased from 1.8 to 5.8 t/ha over five decades (Table 6). While yield and labor productivity gradually increased right up to 2012, net profit and N use efficiency have stagnated since 1980 and 1975, respectively. Higher N use efficiency in 1967 than commonly observed values (40–70 kg yield/kg N; Dobermann, 2005) was due to lower N application rate, and is indicative for soil N mining. Using the same data source as Moya et al. (2015); Laborte et al. (2012) show that the distribution of yields was positively skewed in the late 1960s, implying that there were more farmers getting low yields (subsistence level) with only a few farmers achieving high yields. The distribution became less skewed but wider in range after the 1960s. Furthermore, following Calderini and Slafer (1998), we assessed rice yield stability using data from Central Luzon, the Philippines (Moya et al., 2015). Quadratic polynomial regressions between year and yield data in wet and dry seasons were performed (Fig. 2). Relative yield residuals (i.e., the difference between actual and predicted data presented as percentage of predicted data) were used to evaluate trends in yield stability in relative terms. Rice yields were not different between wet and dry seasons until the late 1970s. Thereafter, rice yield was consistently higher in the dry season. However, relative yield residuals were not largely different between the two seasons. In both seasons, there was no clear trend in increased stability over years (reduced relative yield residual). Thus, it is suggested that rice production systems in this region have been highly successful in increasing yield while maintaining relative yield stability.

**Table 6:** Trends in yield, net profit, labor use, and adoption of rice variety and agronomic practices in irrigated lowland rice in the dry season in Central Luzon Loop Survey, 1966–2012 (adapted from Moya et al., 2015).

Factor	Units	1967	1971	1975	1980	1987	1991	1995	1998	2004	2007	2012
No. observations		17	13	14	81	64	58	56	46	71	68	66
Yield (t/ha)	t/ha (mean)	1.8	2.5	2.0	4.4	4.2	4.4	4.8	4.6	4.8	5.2	5.8
Net profit	PHP/ha*	6,273	12,685	−7,606	20,218	22,987	14,111	24,641	23,109	10,649	28,175	18,110
N use efficiency	kg yield/kg N applied	89	39	56	46	42	43	37	44	44	51	48
Labor productivity	kg/8-h person-days	26	33	20	51	63	74	71	92	92	99	101
Rice variety**												
TV	% of farmers	94	8	7	6	3	0	0	0	0	0	0
MV1	% of farmers	6	92	100	10	9	9	2	13	1	0	0
MV2	% of farmers	0	0	7	89	20	12	7	2	3	1	0
MV3	% of farmers	0	0	0	0	78	83	91	46	6	3	8
MV4	% of farmers	0	0	0	0	0	0	0	43	90	96	92
Hybrid	% of farmers	0	0	0	0	0	0	0	0	0	0	5
Land preparation												
Animal	% of farmers	100	100	79	53	69	98	77	67	58	65	65
Power tiller (2 wheels)	% of farmers	6	0	43	79	88	90	100	93	100	100	100
Large tractor (4 wheels)	% of farmers	47	62	43	9	6	2	9	17	17	18	8
Crop establishment												
Direct seeding	% of farmers	0	0	0	9	48	71	63	54	63	57	30
Transplanting	% of farmers	100	100	100	91	59	33	41	48	41	44	73
Fertilizer application rate												
N fertilizer	kg/ha (mean)	20	64	35	96	100	103	130	104	110	103	119
Frequency of fertilizer application												
1 time	% of farmers	86	93	84	27	63	21	15	16	10	4	10
2 times	% of farmers	14	0	16	67	32	55	56	53	44	49	52
3 times	% of farmers	0	7	0	6	5	24	25	27	41	43	28
> 3 times	% of farmers	0	0	0	0	0	0	4	5	4	4	10
Labor use												
Land preparation	8-h person-days/ha	15	14	16	13	14	13	14	10	11	10	10
Crop establishment	8-h person-days/ha	26	24	38	30	18	11	13	12	11	12	18
Crop care	8-h person-days/ha	10	17	18	12	6	5	6	3	5	4	4
Harvesting and threshing	8-h person-days/ha	19	22	25	32	28	30	34	25	26	26	25
Total	8-h person-days/ha	70	76	98	86	67	59	68	50	52	53	57

* PHP 1 (Philippine peso) = US$ 0.020 (11 Nov 2019).

** MV1 refers to the first generation of modern varieties (MV) released from the mid-1960s to the mid-1970s: C4 developed by the UP College of Agriculture (now UP Los Baños), IR5 to IR34 developed by the International Rice Research Institute (IRRI), and the varieties released by the Bureau of Plant Industry; MV1 were potentially higher-yielding than the traditional varieties (TV). MV2 or the second generation of modern varieties were released from the mid-1970s to the mid-1980s; they were characterized by yield stability by incorporating multiple pest and disease resistances and shorter growth duration: varieties IR36 to IR62. The third generation of MVs (MV3) refers to varieties released from the mid-1980s to the mid-1990s: IR64 to IR74, and PSBRc2 to PSBRc74; these have better grain quality and are adapted to direct-seeding; they are not superior to MV2 in terms of yield. MV4 are those varieties released after 1995, including RC varieties released by the Philippine Rice Research Institute (PhilRice); some of these varieties are more adaptable to harsh environments, such as the drought-resistant varieties and submergence-tolerant varieties. In 2001, the Philippine Government introduced hybrid rice; however, adoption has been low, partially due to problems of seed supply and the quality of the rice (Moya et al., 2015).

### 4.9 Use of data from sub- or national statistics

In the scaling stage, data from sub- or national statistics could be used for assessing agronomic gain, although they are rarely disaggregated by sex. We provide here a case from irrigated lowland rice in Uruguay (Pittelkow et al., 2016; Table 7), as we do not have such data from SSA except for yield. Yield growth rate in Uruguay was 149 kg/ha/year from 1993 to 2004 and is higher than in the cases from the Philippines for a similar period (Table 6). As yield levels of popular rice varieties in the last two decades were similar, Pittelkow et al. (2016) suggest that national yield increase was most likely realized through improved agronomic practices combined with the appropriate selection of each variety for specific regions rather than the higher genetic yield potential of one specific variety over another. N use efficiency did not change in this period, but water productivity increased together with yield increase. Blanco et al. (2010) report that, starting in the mid-1990s, the majority of farmers adopted improved agronomic practices. Specifically, the proportion of rice area under drill-seeding, N and P application at the beginning of the season, N top-dressing, and use of herbicide increased to more than 90 % by around 2010. Together with these practices, earlier planting and early season irrigation could have contributed to increased yield and water productivity, and higher N-use efficiency (Tseng et al., 2021). This case also clearly showed that despite the achievement of high yield through agricultural intensification, negative consequences on the environment were avoided. Total energy consumption has been reduced over years through introduction of conservation and no-tillage agriculture.

**Table 7:** Trends in yield, N fertilizer rate, energy consumption, water input, agrochemical contamination risk, and carbon footprint of irrigated lowland rice production over 1994–2013 in Uruguay (adapted from Figs. 2 to 4 of Pittelkow et al., 2016).

	1994	2004	2013
Yield (t/ha)	4.6	6.6	7.8
N use efficiency (kg yield/kg N applied)	100	125	100
Water productivity (kg/m^3^)	0.3	0.5	0.55
N fertilizer rate (kg/ha)	45	55	78
Total energy consumption (GJ/ha)	21	17	17
Water input (1000 m^3^/ha)	15	15	15

## 5 Key challenges and opportunities for assessment of agronomic gain

Based on our case studies in previous sections that mostly focused on rice in SSA, we found major challenges in the assessment of agronomic gain (Table 2). They include differentiating agronomic gain from genetic gain, unreliable in-person interviews, and assessment of some KPIs at a larger scale. These challenges are more general and could be applicable to other crops and regions as well. In addition to these, we also highlighted the key challenges related to data limitations in rice agronomy research in SSA. In the following sub-section, we discuss these challenges and how to overcome them in future research.

### 5.1 Differentiating agronomic gain from genetic gain

As discussed in Section 4, in discovery (baseline and diagnostic surveys), pilot, and scaling stages, it is often difficult to differentiate agronomic contribution from variety effect. To overcome this issue, Horie et al. (2005) and Pittelkow et al. (2016) assumed that variety effect on farmers’ yield was marginal based on estimation of variety effect using a small data set of previous field trials. This approach could be reasonable, and can be applied to other conditions where farmers have adopted advanced agronomic practices, especially with relatively uniform environmental and management conditions (e.g., irrigated lowland rice). When it comes to countries having large potential agronomic gain in yield and large variation in crop-growing environments in the target environment, it is essential to conduct multi-environment trials in farmers’ fields to predict variety adaptation to different environmental and management conditions in proof of concept stage (Atlin et al., 2017). Such information can help quantify the main G, M, and E effects, and their interactions; identify key environmental and management conditions under which new improved varieties perform well; and quantify agronomic gain in yield.

Using advanced sampling frames based on GIS information on key factors that could govern yield and yield stability, such as weather and soil data, representative field sites for multi-environment trials can be identified with reduced biases (Edreira et al., 2018). This framework could significantly reduce the number of trial sites, improve cost-efficiency, and facilitate rapid evaluation and scaling of the agronomic practices. Site characterization of on-farm trials at scale is now possible through handheld sensors and apps (e.g., landpotential.org) that link to remote sensing data and identify site soil and environmental properties as shown recently in Malawi (Ewing et al., 2021). Furthermore, use of crop simulation models together with data on weather, soil, and agronomic practices, as well as data collected from multi-environment trials, could help more precisely identify target environment and potential impact on KPIs (van Oort et al., 2016).

### 5.2 Unreliable in-person interviews

Data on agronomic gain at field level are often collected through farm records or household recall survey, with far fewer efforts to collect sex-disaggregated data at plot level (Kilic et al., 2015). Sometimes, farmers are asked to record their interventions by themselves, and farmers receive regular visits by enumerators who monitor their records (SRP, 2019). In other cases, enumerators visit farmers to ask for the required information at a few times during the period from land preparation to post-harvest (Niang et al., 2017). Self-reported data by smallholder farmers or surveyed data are often inaccurate and the accuracy of methods used to assess the agronomic gain needs to be improved (Lobell et al., 2019). Field size can be verified through direct measurement with a measuring tape or calculation of area on a map. Furthermore, rapid estimation of yield could be possible with use of simple sensors (e.g., smartphone camera), remote-sensing technology together with crop simulation model, machine learning, and artificial intelligence (Saito et al., 2013; Boschetti et al., 2017; Setiyono et al., 2019). Soil health could be indirectly determined with non-destructive mobile devices, if the accuracy of estimation is improved further (e.g., Chatterjee et al., 2009). For agronomic gain in other KPIs (profitability, labor productivity, nutrient use efficiency), data still needs to be collected through farm records or household (ideally, within household) recall survey. Here, recent innovations in electronic surveys (e,g, opendatakit.org) could improve the reliability and rapid data collection capacity for improved rigor in this exercise.

However, some of parameters required for calculation of KPIs such as N fertilizer application rate and labor use could be also indirectly determined if there are significant relationships between those parameters and other parameters that can be more easily obtained through interviews. For example, in the case for the Philippines in Section 4.8, it was found that there are significant relationships between N fertilizer application rate and % of farmers applying N once (r = −0.85, p < 0.01), labor use for land preparation and % of farmers using power tiller (r = −0.65, p < 0.05), and labor use for crop establishment and % of farmers planting rice with transplanting (r = 0.91, p < 0.01). Such relationships can be used to estimate N fertilizer application rate and labor use for land preparation and crop establishment, as these self-reported data by smallholder farmers are often inaccurate.

### 5.3 Difficulty in assessing some KPIs at a larger scale

We recognize that the direct monitoring of water input and water productivity is not possible except for on-station trials in proof of concept stage. Thus, it is essential to establish and validate their relationships with parameters that can be easily assessed through interviews or field observations. This could also significantly reduce monitoring cost. For example, water inputs could be roughly estimated using amount of rainfall, number of irrigation regimes, and depth of irrigation (Devkota et al., 2019).

Analyzing relationships among the indicators could also help reduce the number of indicators to be measured, but still assessed for a full assessment of success based on all KPIs together. For example, as shown in 4.1 and 4.9, water productivity can be highly correlated with yield. In this case in Philippines in 4.8, rice yield, net profit, and labor productivity were positively correlated (r = 0.67–0.94, p < 0.05), but not with N-use efficiency. Thus, for reducing time and cost for monitoring, for some years, it could be possible to monitor yield and N-use efficiency more frequently, and assessment of these three indicators can be done only from time to time.

Furthermore, rapid advances are being made in remote-sensing technology for estimating various parameters such as crop area, weather data, soil fertility, cropping season, water availability, and yield (Saito et al., 2013; Boschetti et al., 2017; Setiyono et al., 2019; Ewing et al., 2021). Further research is needed to know if this technology can assess adoption of improved agronomic interventions and their impact on agronomic gain in KPIs.

### 5.4 Potential use of indirect assessment for KPIs via agronomic practices

If agronomic practices and some KPIs are highly correlated, assessing KPIs can be indirectly done through collecting data on agronomic practices in scaling stage. For example, the two studies from the Philippines and Uruguay reported on in this paper consistently show that mechanization for timely planting and N fertilizer management are important contributors to agronomic gain over years. This is supported by the case in Japan (Horie et al., 2005). Furthermore, the case for mechanization is clearly further supported by the counter-example in irrigated lowland rice cultivation in the Senegal River valley, Senegal. Yield in the panel survey in this area was stagnant over the period 2002–2010, where poor or limited access to tractors for land preparation resulted in delayed planting that significantly reduced yield (Tanaka et al., 2015). From these cases, one could collect information on mechanization for timely planting and N fertilizer management for rapid indirect assessment for yield.

Our above cases are from irrigated lowland rice. Monitoring for agronomic gain might be more challenging in rainfed rice systems in SSA (Table 3), which have larger spatial–temporal variability in yield, for example. Especially in harsh environments (drought- or flood-prone conditions), farmers’ agronomic practices can differ across years. When drought occurs before planting, planting cannot be done on recommended dates, and farmers might not want to invest in applying fertilizer in that year as fertilizer can be wasted in drought. In another case, with good rainfall at the beginning of the season, farmers might decide to invest a lot in fertilizer application, but then sudden flooding could result in crop failure. Here, simple comparisons between yield and agronomic practices would not work under such conditions. In this case, additional parameters such as Information on farmers decision-making, access to crop insurance (if farmers have this, they might not worry so much about crop failure), access to supplemental irrigation and weather data would be needed for linking agronomic practices and other data with agronomic gain.

### 5.5 Limited data on rice agronomic gain KIPs in SSA

Based on our review of the literature for this paper, rice agronomy research is lacking in the following areas: (i) the quantification of potential agronomic gain at the discovery stage, except for yield; (ii) previous studies mainly focus on agronomic gain KPIs in the proof of concept stage; and (iii) there are limited studies including panel studies available in pilot and scaling stages. Establishment of panel surveys for monitoring progress toward impact on KPIs is therefore necessary, as there are limited data in subnational or national levels in SSA. Long-term investments by SSA governments and donors are essential for ensuring that such monitoring can be sustained.

We recognize that the development and promotion of agronomic practices has been the major focus in SSA to enhance rice productivity and address food insecurity (Tollens et al., 2013). This focus presumably explains why most previous studies in the poof-of-concept stage assessed yield only or yield and a few other indicators. However, with the establishment of EiA 2030, coupled with the KPIs’ strategic linkages with impact areas of the One CGIAR research and innovation strategy 2030 (CGIAR System Office, 2021) and the proposals in this paper to circumvent the challenges for assessing KPIs will facilitate the assessment of the KPIs to determine the tradeoffs and synergistic effects when improved agronomic practices are tested.

Furthermore, long-term trials with agronomic innovations especially for rainfed rice systems are needed for assessing yield stability as well as other KPIs. Unique insights may be derived from linking together long-term trials with multi-locational on-farm trials (Snapp et al., 2019).

## 6 Conclusions

Food production must increase substantially without negative impact on the environment to meet the global food security needs of the future in a sustainable manner. Large yield gaps and low yield stability for rice exist in many places, especially in SSA. There is, however, a great opportunity for closing yield gaps and enhancing sustainability through agronomic practices. This study presented a definition of agronomic gain and described different approaches for its assessment in four different stages. Major challenges to assess agronomic gain include differentiating agronomic gain from genetic gain in pilot and scaling stages, unreliable in-person interviews, assessment of some of the KPIs at large scale, and data limitations in different stages. We suggest to assess variety (G) × agronomic practice (M) × environment (E) interaction through multi-environment trials in proof of concept stage, which allow us to predict crop response to agricultural practice, variety, and their interaction under different environmental conditions and social contexts within the target environment. M × E interaction should be carefully analyzed to understand under which conditions higher or lower agronomic gain is obtained. Furthermore, we suggest to develop novel approaches for assessing KPIs, through development and introduction of remote-sensing technology and/or mobile devices that can estimate KPIs (e.g., yield, soil health) and establishment of empirical relationships among KPIs or between agronomic practices and KPIs for indirect assessment. Implementing panel surveys are essential for monitoring progress towards impact along with establishing long-term trials on improved agronomic practices for assessing yield stability as well as other KPIs, especially for rainfed rice systems. These proposed research areas would facilitate developing, evaluating, and scaling new innovations with proposed agronomic gain assessment to achieve their widespread adoption by different end users based on their needs and preferences.

## Declaration of Competing Interest

The authors report no declarations of interest.
